# PCL-gelatin honey scaffolds promote *Staphylococcus aureus* agrA expression in biofilms with *Pseudomonas aeruginosa*

**DOI:** 10.3389/fmicb.2024.1440658

**Published:** 2024-09-03

**Authors:** Genevieve M. Hilliard, Thomas Stephens Wilkinson, Llinos G. Harris, Rowena E. Jenkins, Laurie P. Shornick

**Affiliations:** ^1^Department of Biology at Saint Louis University, St. Louis, MO, United States; ^2^Biomedical Sciences at University of Swansea, Swansea, Wales, United Kingdom

**Keywords:** biofilm, honey, bacteria, wound care, ESKAPE pathogens

## Abstract

**Introduction:**

Bacterial infection and biofilm formation contribute to impaired healing in chronic diabetic wounds. *Staphylococcus aureus* and *Pseudomonas aeruginosa* are found in human diabetic wound biofilms. They may develop antibiotic resistance, increasing the urgency for alternative or complementary therapies. Diabetic wound healing may be improved with the use of biomedically engineered scaffolds, which can also serve as delivery systems for antibacterial compounds. Manuka honey is a potent antibacterial and wound care agent due to its high osmolarity, low pH, and constituents (such as methylglyoxal). Honey exhibits bacteriostatic and bactericidal effects, modulates the expression of biofilm forming genes, and restores antibiotic susceptibility in previously drug resistant pathogens.

**Methods:**

In this study, we created a dermal regeneration template (DRT) composed of polycaprolactone-gelatin (PCL-gelatin) and Manuka honey to retain honey in the wound and also provide a scaffold for tissue regeneration.

**Results and discussion:**

Soluble Manuka honey inhibited the planktonic and biofilm growth of both *S. aureus* (UWH3) and *P. aeruginosa* (PA14) co-cultures. Manuka honey embedded PCL-gelatin scaffolds did not exhibit bacteriostatic or bactericidal effects on cocultures of UHW3 and PA14; however, they promoted the expression of *AgrA*, a gene associated with dispersal of *S. aureus* biofilms.

## Introduction

Diabetes Mellitus afflicts more than 430 million people worldwide, and is associated with impaired wound healing, which can be life-threatening. Approximately 25% of patients with diabetes will develop a chronic ulcer (Kalan and Brennan, 2019). Current first line treatments for diabetic foot ulcers and infections often fail to promote full wound closure and frequently result in lower limb amputation (Brennan et al., 2016; Martins-Mendes et al., 2014; Walsh et al., 2016). After amputation, a patient’s 5-year mortality reaches 50%, causing healthcare practitioners to label ulcers as a silent epidemic (Brennan et al., 2016; Martins-Mendes et al., 2014; Walsh et al., 2016). The incidence of chronic wounds is expected to steadily increase as diabetes becomes more common worldwide (Sen et al., 2009). Furthermore, antimicrobial resistance has rendered many standard antibiotic regimens ineffective and the CDC has declared it to be one of the greatest threats of our age (Services USDoHaH, 2019).

A significant clinical challenge in treating chronic diabetic wounds is that they often contain biofilms composed of drug resistant *Staphylococcus aureus* (*S. aureus*) and *Pseudomonas aeruginosa* (*P. aeruginosa*) (Brem and Tomic-Canic, 2007; Kalan and Brennan, 2019; Sharma et al., 2019). Biofilms are communities of bacteria that adhere to each other and to a surface. Formation of these complex structures may increase the antibiotic resistance of the bacteria. Bacterial quorum sensing systems (QSS) provide the cell-to-cell communication needed to form biofilms (Pena et al., 2019; Rutherford and Bassler, 2012). The wound pathogens *S. aureus* and *P. aeruginosa* are both associated with delayed healing and infection in acute and chronic wounds and are the leading causes of diabetic foot infections (Abdulrazak et al., 2005; Bowler et al., 2001). *S. aureus* and *P. aeruginosa* have been shown to interact in biofilms, which may promote early colonization and pathogenicity of wounds (Alves et al., 2018; Bowler et al., 2001; Nguyen and Oglesby-Sherrouse, 2016). For *S. aureus*, the accessory gene regulator (agr) QSS regulates the phenol soluble modulin peptide to form biofilms (Tan et al., 2018). Biofilm formation in *S. aureus* is inhibited by the *agr* system of quorum sensing genes (Boles and Horswill, 2008; Vuong et al., 2000). In contrast, *P. aeruginosa* utilizes a hierarchical QSS consisting of *Las, pqs and rhl* to coordinate virulence genes and biofilm formation (Lee et al., 2013).

Manuka honey has emerged as a promising treatment for bacterial infections. Named by the Maori tribe, Manuka is the common name for the shrub *Leptospermum scoparium*, which is found in Southeast Australia and New Zealand (Lu et al., 2019; Mandal and Mandal, 2011). The shrub is pollinated by *Apis Mellifera*, and the nectar collected from the shrub is used to make a unique honey (Carter et al., 2016). Like most types of honey, Manuka has a low pH and high osmolarity, which inhibits pathogen growth. It has become the gold standard for wound care due to the presence of methylglyoxal, a potent antibacterial agent that exerts its action by cross linking proteins (Roberts et al., 2015). Manuka honey inhibits the growth of Methicillin-resistant *S. aureus* by inhibiting cell division and reduces the expression of genes such as Stress Protein A, which confers adaptive resilience in hostile environments (Jenkins et al., 2011a, 2011b). Expression of virulence genes, resistance genes, and quorum sensing genes in bacterial species such as *S. aureus* and *P. aeruginosa* are also reduced in response to Manuka honey (Ahmed and Salih, 2019; Jenkins et al., 2014; Wang et al., 2012). *In vitro* experiments demonstrate that Manuka honey can even restore antibiotic susceptibility to previously drug-resistant organisms (McLoone et al., 2016a). Importantly, Manuka honey may inhibit the growth of *S. aureus* and *P. aeruginosa* biofilms (Alandejani et al., 2009).

Biomedically engineered scaffolds support wound healing by providing a structure for cells to utilize during reepithelialization. Scaffolds can be customized by embedding compounds to promote healing and inhibit microbial growth. We previously developed a dermal regeneration template (DRT) composed of polycaprolactone (PCL) and varying concentrations of Manuka honey (1, 5, 10, and 20% w/v) (Hilliard et al., 2019). We demonstrated that the PCL-honey scaffolds allowed for cellular infiltration, proliferation, and endothelial tube formation *in vitro*. In a murine model of excisional wound healing, the DRT also facilitated cell infiltration and prevented rapid wound contraction in both wild type and diabetic mice (Hilliard et al., 2019). In this study, we enhanced the bioactive property of the DRTs by incorporating gelatin. Gelatin is a denatured form of collagen with high biocompatibility and healing effects. *In vivo* wound studies demonstrated that scaffolds containing gelatin promote granulation tissue formation, re-epithelialization, and angiogenesis (Akhavan-Kharazian and Izadi-Vasafi, 2019; Hsu et al., 2019; Jang et al., 2018; Jinfeng et al., 2019; Nikpasand and Parvizi, 2019).

The aim of this study was to determine the effect of Manuka honey treatment on planktonic bacteria and established biofilms containing the wound pathogens *S. aureus* (UHW3) and *P. aeruginosa* (PA14). Bacteria were treated with honey directly or embedded in a dermal regeneration template. PCL-gelatin-honey scaffolds containing a 10% or 20% honey solution (w/v) were synthesized and tested *in vitro* with the wound pathogens *S. aureus* (UHW3) and *P. aeruginosa* (PA14). We hypothesized that the honey scaffolds would inhibit the planktonic and biofilm growth of UHW3/PA14 co-cultures and reduce the expression of genes involved in biofilm formation. PCL, gelatin, and Manuka honey are three materials used extensively in wound healing research. This is the first study to test the antibacterial effects of a PCL-gelatin-Manuka honey scaffold on co-cultures of *S. aureus* and *P. aeruginosa*.

## Methods

### Bacterial strains and antibiotics

*S. aureus* (UHW3) and *P. aeruginosa* (PA14) clinical isolates obtained from a diabetic ulcer and burn wound, and reference strains *S. aureus* NCTC8532 and *P. aeruginosa* NCTC13437, were used in this study. All bacterial strains were obtained from the National Collection of Type Cultures (NCTC). Antibiotics for microbiology testing were purchased from Thermo-Fisher.

### Bacterial cell culture

Bacterial stocks were streaked onto Mueller-Hinton agar (MHA, Sigma). After 24 h, colonies were inoculated in sterilized brain-heart-infusion (BHI, Sigma) broth and diluted to 0.08–0.1 O.D. at 600 nm.

### Colony forming units

To determine colony forming units (CFU’s/mL), liquid suspensions of bacterial isolates were diluted 10-fold, and 20 μL of each dilution was plated on Mannitol salt agar (MSA), Pseudomonas isolation agar (PSA) or BHI plates. The CFU/mL were determined by multiplying the number of colonies X the dilution factor X 50. These culture conditions and determination of CFU/mL were utilized for experiments described.

### Minimum inhibitory concentration (MIC)

Bacterial strains were brought to 0.08–0.1 O.D at 600 nm, then diluted 1:100 in sterilized brain heart infusion (BHI) broth, and 100 μL of this suspension was added to a 96 well plate. Sterile medical grade Manuka honey (Medihoney; Derma Sciences) was diluted aseptically in sterilized BHI broth. The concentrations of honey used were based on previous studies demonstrating honey inhibition of bacterial growth: 52, 48, 44, 40, 36, 32, 28, 24, 20, and 16% (w/v) (Maddocks et al., 2013). Next, 100 μL of each concentration was added to separate wells of the 96-well plate, thus generating the final honey concentrations in each respective well: 26, 24, 22, 20, 18, 16, 14, 10, and 8% (w/v) (*n* = 3). Plates were incubated at 37°C without shaking. The bacterial density was recorded with a SPECTROstar® Nano spectrophotometer at 600 nm at time zero for each well and again after 24 h. The planktonic cell suspension was removed after 24 h and plated on BHI plates according to the colony forming units protocol specified under “Bacterial cell culture” (*n* = 3). The MIC was determined as the lowest concentration of honey which showed no growth on the 96-well plate.

### Minimum biofilm inhibitory concentration (MBIC)

Bacterial strains were brought to 0.08–0.1 O.D at 600 nm, then diluted 1:100 in sterilized brain heart infusion (BHI) broth, and 100 μL of this suspension was added to a 96 well plate. Subsequently, the following concentrations of medical grade Manuka honey (Medihoney) were generated aseptically by diluting in sterilized BHI broth: 52, 48, 44, 40, 36, 32, 28, 24, 20, and 16% (w/v) (Maddocks et al., 2013). Next, 100 μL of each concentration was added to separate wells of the 96-well plate, thus generating the final honey concentrations in each respective well: 26, 24, 22, 20, 18, 16, 14, 10, and 8% (w/v) (*n* = 3). Plates were incubated at 37°C without shaking. After 24 h incubation, the broth was removed and the bacteria were washed twice with 200 μL of PBS. Each well was filled with 200 μL of methanol and incubated at room temperature for 30 min. The residual methanol was removed from each well and the plate was air dried for 30 min at room temperature. Next, 200 μL of 0.1% crystal violet was added to each well and incubated at room temperature for 15 min. Crystal violet was then removed and the wells were washed twice with PBS. Finally, 200 μL of 7% glacial acetic acid was added to the wells and incubated for 15 min at room temperature. Crystal violet absorbance was measured at 590 nm. In separate plates, the attached biofilm was scraped off the bottom of the plate after 24 h, resuspended in 200 μL of PBS, and plated on BHI plates according to the colony forming units protocol specified under “Bacterial cell culture” (*n* = 3). MBIC was taken as the lowest concentration of honey which showed no growth on the BHI plate.

### Minimum biofilm eradication concentration (MBEC)

The MBEC assay differed from the MBIC assay in that bacteria were grown for 24 h in wells prior to the addition of manuka honey. In this method, the concentration of honey required to kill or inhibit the growth of already established biofilms was measured. Bacterial strains were brought to 0.08–0.1 O.D at 600 nm, then were diluted 1:100 in sterilized BHI broth and 200 μL of the bacterial cell suspension was added to a 96 well plate (*n* = 3). After 24 h, the broth was removed from the wells and 200μL of Manuka honey, resuspended aseptically in sterilized BHI broth, was added to separate wells to generate the following final concentrations: 64, 32, 16, 8, 4, and 2% (w/v). Previous studies demonstrated a requirement for higher concentrations of honey to inhibit biofilm growth (Maddocks et al., 2013). Therefore, the highest possible concentration of honey achievable (64%) was used for biofilms that were already established. Plates were incubated at 37°C without shaking. After 24 h, each well was washed twice with 200 μL of PBS. Each well was then filled with 200 μL of methanol and incubated at room temperature for 30 min. The methanol was removed from the wells and the plate was air dried for 30 min at room temperature. Then 200 μL of 0.1% crystal violet was added to each well and incubated at room temperature for 15 min. The crystal violet was removed and each well was washed twice with PBS. Crystal violet was dissolved with 200 μL of 7% glacial acetic acid for 15 min at room temperature and absorbance was measured at 590 nm. In separate plates, the attached biofilm was scraped off the bottom of the plate after 24 h of incubation with honey, resuspended in 200 μL of PBS, and plated on BHI plates according to the colony forming units protocol specified under “Bacterial cell culture” (*n* = 3). The MBEC was taken as the lowest concentration of honey which showed no growth on the BHI plate.

### Scaffold fabrication

The PCL-gelatin-honey scaffolds were generated by combining polycaprolactone (Sigma Aldrich), fish gelatin (Sigma Aldrich), and Manuka honey (ManukaGuard, MGO 400). Specifically, 0.25 g PCL and 0.25 g gelatin were dissolved overnight in 4.5 mL of 1,1,1,3,3,3-hexafluoro-2-propanol (HFP) (Oakwood Chemical). After 18–24 h, 500 μL of Manuka honey was added to the PCL-gelatin mixture to create a 4% PCL, 4% gelatin, and 10% honey (w/v) solution. The honey solution was sonicated for 30 min in a room temperature ultrasonic water bath. Five milliliters of the honey solution was loaded into a 5 mL syringe tipped with a blunted 18-gauge needle (PrecisionGlide, Becton Dickinson). Additional solution was loaded into the syringe and extruded, if necessary, to achieve a scaffold thickness of 0.3 mm – 0.6 mm. Briefly, the syringe was placed in an electrospinning device and high voltage (20-25 kV) was applied using a high-voltage DC power supply. The solution ejected onto a grounded, moving aluminum circular disk at a distance of 10–13.5 cm from the needle with a flow rate of 1.5–2.5 mL/h. Parameters were adjusted according to the efficiency of extrusion throughout the process of electrospinning. Once the solution was expended, the honey scaffold was stored in a paraffin sealed petri dish at −20°C. Circular punches of the scaffold were generated with a 10 mm punch biopsy tool. Control scaffolds without honey were generated in the same manner as described above. They were placed in a petri dish and stored in a desiccator at room temperature.

### Scaffold-biofilm co-culture

UHW3 and PA14 were seeded together into a 24 well plate at a ratio of 250:1. Previously this ratio has been shown to allow for growth of *S. aureus* with *P. aeruginosa* cultures, preventing *P. aeruginosa* from outcompeting *S. aureus* growth (Woods et al., 2018). A control scaffold without honey, or a scaffold composed of 10% or 20% Manuka honey solution (w/v), was added immediately to the wells. After 24 h at 37°C, the planktonic bacteria were removed and washed gently with 0.5–1 mL PBS. The planktonic bacteria were centrifuged at 13,000 x g for two minutes. The supernatant was discarded, and the pellet resuspended in 500 μL of PBS. The remaining biofilm was resuspended in 500 μL of PBS using a cell scraper (Starlab). CFUs/mL were determined. Additionally, the scaffolds were each cut in half, and each half was plated on either MSA or PSA to determine growth on the scaffold. After 24 h incubation, the area of growth around the scaffold was determined by ((TA-AS)/TA)*100 where TA is the total area of bacterial growth and AS is the area of the scaffold.

### Bacterial RNA isolation

UHW3 and PA14 were seeded together into a 24 well plate at a ratio of 250:1. A control scaffold without honey, or a scaffold composed of 10% or 20% Manuka honey (w/v), was added immediately to the wells. After 24 h, the planktonic bacteria were removed and washed gently with 0.5–1 mL PBS. The planktonic bacteria and the washed cells were centrifuged at 14,000 rpm for five minutes. The supernatant was removed, and the pellet resuspended in 500 μL of PBS and 1 mL of RNAprotect® (Qiagen). The tubes were gently inverted and placed at room temperature for five minutes. The bacteria were centrifuged at 5,000 rpm for 10 min. The supernatant was removed and the pellet stored at −80°C. The biofilm was re-suspended with a cell scraper directly in 200 μL of a cell lysis buffer containing lysostaphin (5 mg/mL, Sigma from *Staphylococcus staphylolyticus*), mutanolysin (10 KU/mL, Sigma from *Streptomyces globisporus*), lysozyme (10 mg/mL Thermo Scientific from hen egg white), and proteinase K (20 mg/mL Qiagen) in TE buffer. The biofilm and scaffold were transferred to a 2 mL centrifuge tube and incubated for one hour at 37°C on a rotating wheel. RNA was isolated using an SV-total RNA isolation system from Promega according to the manufacturer’s instructions. RNA samples were treated with rigorous DNAse treatment according to manufacturer’s instructions (Thermofisher TURBO DNA-*free*™ kit).

### Gene expression

The RNA concentration (ng/μL) was determined with a NanoDrop One (Thermofisher scientific). Two-hundred nanograms of RNA from each sample was converted into cDNA in a S1000 Thermal Cycler (Biorad) using a qPCRBIO cDNA synthesis kit (PCR biosystems). Unless noted that the sequences came from a published paper, all sequences were generated from Primer3 Input using the annotated genome for each strain. The specificity of the primers within co-cultures was determined through melting curve analysis with SYBR green (Meridian Bioscience SensiMix SYBR No-ROX Kit). qPCR was performed in a Quant5 thermocycler with the following protocol: hold at 50°C for 2 m, hold at 95°C for 2 m, then repeat the following 40X: 95°C for 15 s, 60°C for 15 s, and 72°C for 1 m. Melt curve analysis was performed after each qPCR run at the following: 95°C for 15 s, 60°C for 1 m, and 95°C for 15 s. Relative gene expression of AgrA and LasR was calculated by the Delta–Delta CT method using 16S as the housekeeping gene utilizing appropriate primers (Livak and Schmittgen, 2001; Table 1).

**Table 1:** PCR primer sequences.

Gene	Forward primer sequence (5'→3')	Reverse primer sequence (5'→3')
AgrA	CCTATGGAAATTGCCCTCGC	TCCGTAAGCATGACCCAGTT
16S staph	TGCACATCTTGACGGTACCT	CGTGGAGGGTCATTGGAAAC
LasR	AGCGACCTTGGATTCTCGAA	CGTACTGCCGATTTTCTGGG
16S pseud	TCTTCGGACCTCACGCTATC	GGCAGCAGTGGGGAATATTG

### Statistical analysis

All graphs show the mean ± standard deviation. Statistical analysis was performed using a one-way ANOVA with GraphPad Prism. The significance level was set at *p* < 0.05.

## Results

### Manuka honey inhibits planktonic growth of *Staphylococcus aureus* and *Pseudomonas aeruginosa*

To characterize *S. aureus* (UHW3) and *P. aeruginosa* (PA14), zone of inhibition and minimum inhibitory concentration, assays were conducted with a panel of EUCAST antibiotics. Susceptibility and resistance were determined using the EUCAST Clinical Breakpoint Tables v. 14.0, valid from 2024-01-01. UHW3 and PA14 were compared to type strain controls (NCTC8532 and NCTC13437, respectively). UHW3 was resistant to ciprofloxacin, erythromycin, and cefoxitin (a proxy for methicillin resistance), while control strain 8,532 was resistant to all antibiotics tested (Supplementary Figure S1A). Control strain 13,437 was resistant to all antibiotics, while PA14 was susceptible to all antibiotics tested (Supplementary Figure S1B). Because UHW3 exhibited greater resistance to cefoxitin (and therefore methicillin), that strain was selected over the reference strain 8,532 for further analysis. It required 15% honey (w/v) to inhibit 8,532 and UHW3, and at least 25% honey (w/v) to fully inhibit planktonic growth of PA14. Based on the antibiotic resistance profile and high honey concentrations (30% w/v) required to inhibit strain 13,437, it was removed from further analysis (Supplementary Figure S2).

### Establishment of *Staphylococcus aureus* and *Pseudomonas aeruginosa* biofilms

To mimic the microbiome of a diabetic ulcer, *S. aureus* and *P. aeruginosa* were grown together at a ratio of 250:1 and biofilms were quantified by crystal violet staining and spectrophotometry. There was significantly more planktonic UHW3 compared to planktonic PA14 after 4 and 8 h in co-culture (*p* = 0.0022 and *p* < 0.0001 respectively) (Supplementary Figures S3A,B). However by 24 h, both pathogens are present in equal quantities within the well, both in planktonic and biofilm form (Supplementary Figure S3C). It was determined that seeding at a ratio of 250:1 (UHW3:PA14) would allow for the development of robust biofilms containing both UHW3 and PA14 after 24 h. This ratio and time point was utilized throughout the study.

### High honey concentrations inhibit growth of UHW3 and PA14 strains

**Figure 1:**
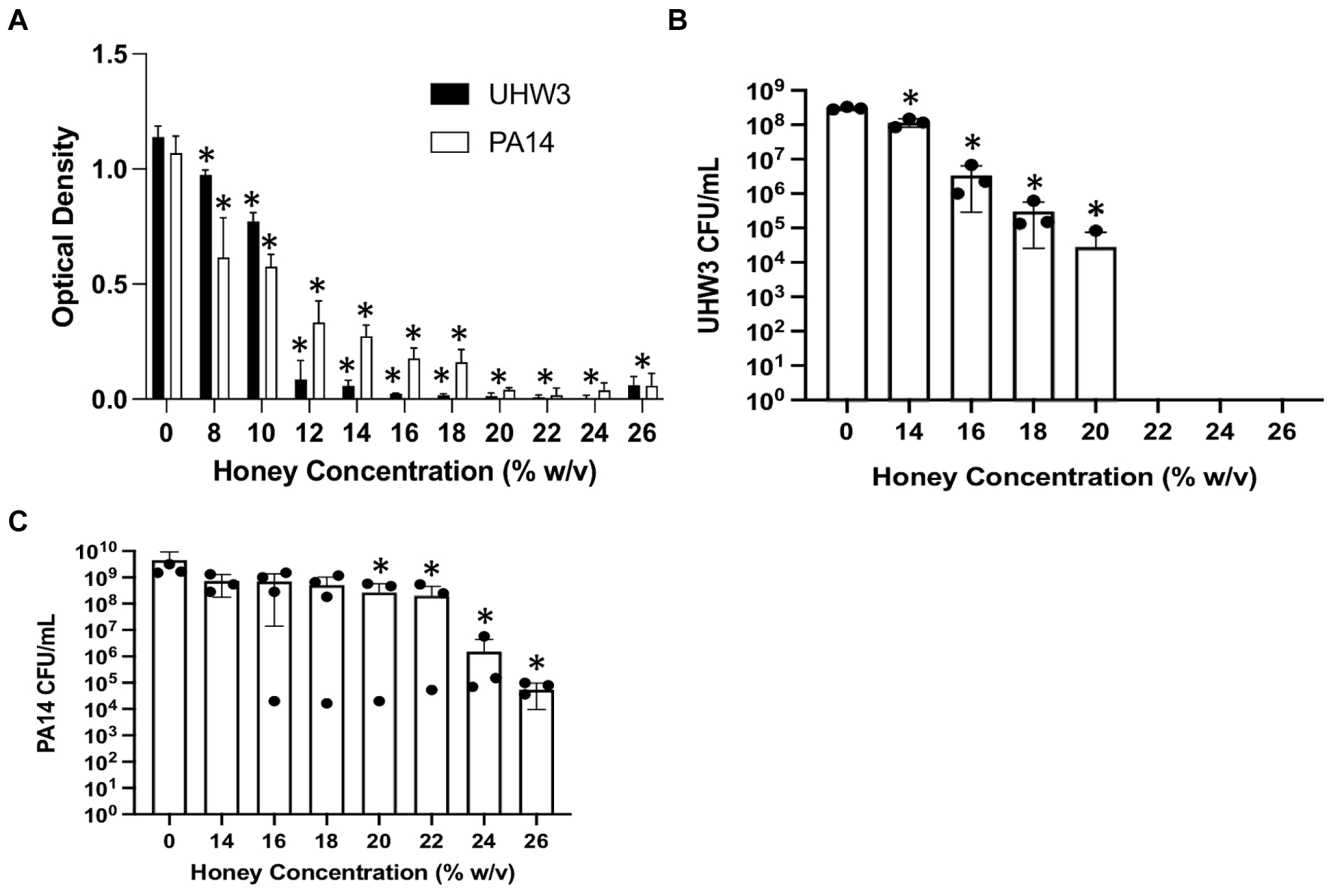
Inhibiting planktonic UHW3 and PA14 requires at least 14% (w/v) and 20% (w/v) honey. **(A)** UHW3 and PA14 were cultured with honey concentrations ranging from 8% (w/v) to 26% (w/v). The OD was obtained to determine the concentrations of honey required to inhibit the growth of UHW3 and PA14 (*n* = 3). Colony forming units per milliliter were calculated for **(B)** UHW3 (*n* = 3) and **(C)** PA14 (*n* = 3 or 4) to determine the viable colonies still present after incubation at 37°C for 24 h with honey. Error bars are represented as standard deviation. Significance was determined with a one-way or two-way Anova Tukey test. An asterisk indicates a significant difference from the no honey control (*p* < 0.05).

We tested the direct effect of Manuka honey on planktonic bacterial growth. Figure 1A shows that both planktonic UHW3 and PA14 growth declined significantly with increasing concentrations of honey in comparison to the no honey control (*p* < 0.0001 for all comparisons except 0% (w/v) vs. 8% (w/v) UHW3 where *p* = 0.0353). Corresponding colony forming units (Figure 1B) confirmed that there was significantly less growth of planktonic UHW3 with concentrations of 14% (w/v) honey or greater in comparison to the no honey control (*p* < 0.0001) (Figure 1B). At concentrations of 22% (w/v) honey and higher, no viable UHW3 colonies were detected. Similarly, there was significantly less planktonic PA14 growth with 20% (w/v) honey or greater in comparison to the no honey control (*p* = 0.0469, 0.0421, 0.0299, and 0.0298 for 20, 22, 24, and 26% (w/v) respectively) (Figure 1C).

**Figure 2:**
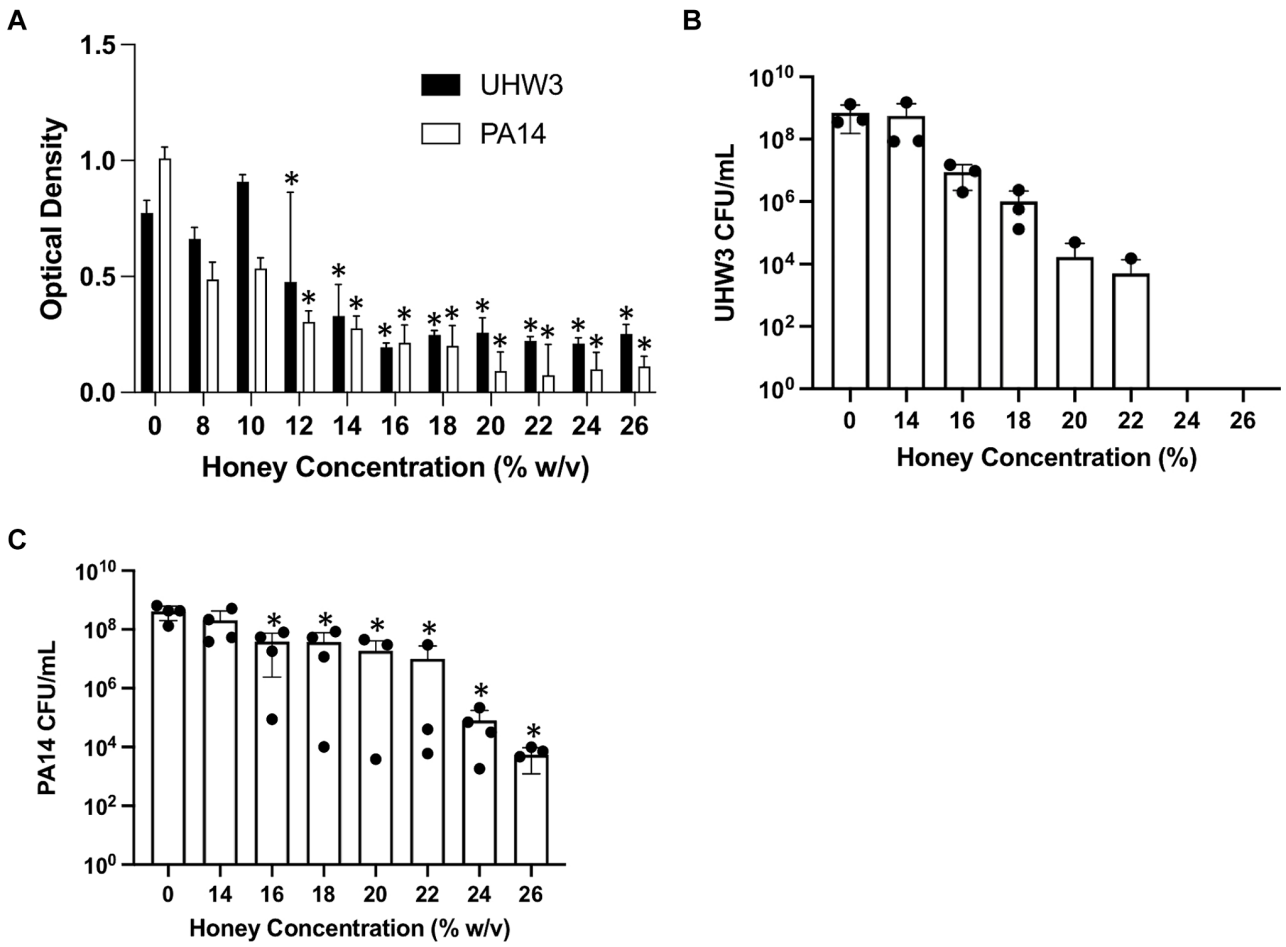
Inhibiting biofilm growth of UHW3 and PA14 requires at least 16% (w/v) honey. **(A)** UHW3 and PA14 were cultured with honey concentrations ranging from 8% (w/v) to 26% (w/v). After 24 h, the biofilms attached to the 96-well plates were stained with crystal violet and the OD was obtained to determine the concentrations of honey required to inhibit biofilm growth (*n* = 3). Colony forming units per milliliter were calculated for **(B)** UHW3 (*n* = 3) and **(C)** PA14 (*n* = 3 or 4) to determine the viable colonies still present after 24 h incubation at 37°C with honey. Error bars are represented as standard deviation. Significance was determined with a one-way or two-way Anova Tukey test. An asterisk indicates a significant difference from the no honey control (*p* < 0.05).

The effect of honey on biofilm formation of the individual bacterial strains UHW3 or PA14 as determined by the crystal violet assay showed a significant reduction with increasing concentrations of honey in comparison to the no honey control (*p* < 0.0001 except for 0% v 12 and 0% vs. 14% (w/v) for UHW3 where *p* = 0.0407 and 0.0002) (Figure 2A). Similarly, counting viable colonies revealed that biofilm growth was inhibited with increasing concentrations of honey. Specifically, there were no detectable colony forming units remaining in UHW3 biofilms with Manuka honey concentrations of 24% (w/v) or higher (Figure 2B). Biofilm growth of PA14 was inhibited at concentrations of 16% (w/v) or higher in comparison to the no honey control (*p* = 0.0022, 0.0022, 0.0012, 0.0023, 0.0007, 0.0007 for 16, 18, 20, 22, 24, and 26% (w/v) respectively) (Figure 2C).

**Figure 3:**
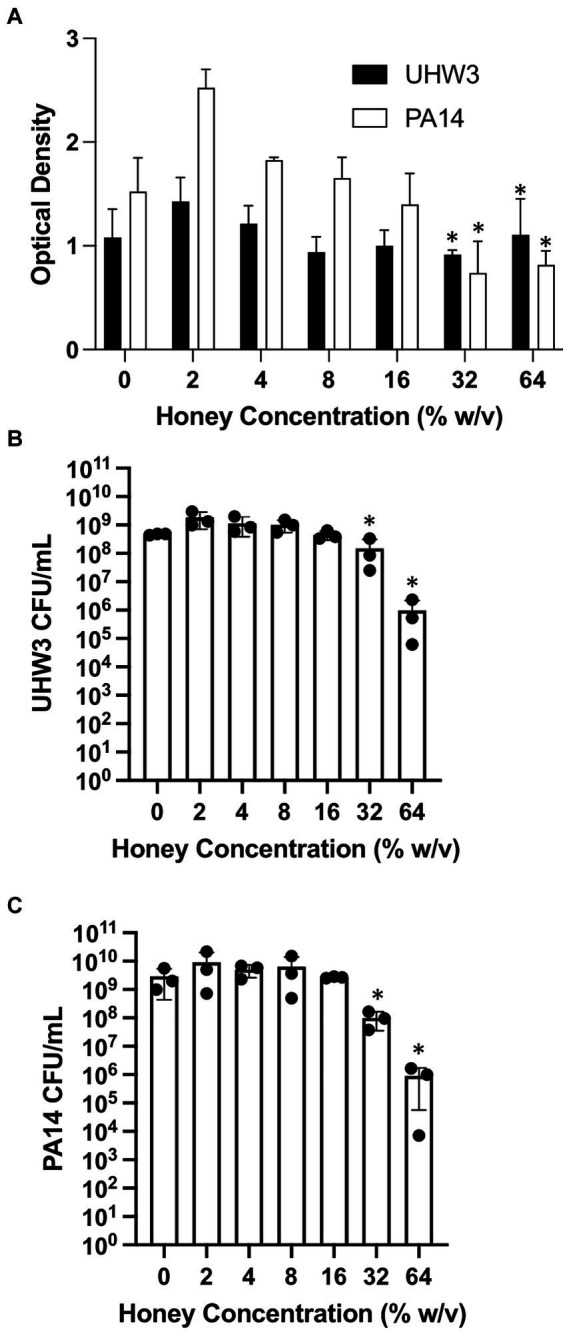
Established biofilms of UHW3 and PA14 are not eradicated with honey. **(A)** UHW3 and PA14 were grown overnight at 37°C to form established biofilms. The biofilms were then exposed to honey concentrations ranging from 2% (w/v) to 64% (w/v) (*n* = 3). Biofilms were stained with crystal violet and the OD was obtained to determine the concentrations of honey required to eradicate biofilm growth. Colony forming units per milliliter were calculated for **(B)** UHW3 (*n* = 3) and **(C)** PA14 (*n* = 3) colony forming to determine the viable colonies still present after 24 h incubation at 37°C with honey. Error bars are represented as standard deviation. Significance was determined with a one-way or two way Anova Tukey test. An asterisk indicates significance (*p* < 0.05).

We next established biofilms containing both *S. aureus* and *P. aeruginosa* as described above (seeded at a ratio of 250:1 for 24 h) and tested the effect of Manuka honey on these biofilms. Biofilm growth was quantified by the crystal violet assay (Figure 3A). For biofilms containing both bacterial strains, growth declined as honey concentrations increased; however, even high concentrations of honey (64% w/v) did not fully eradicate established biofilms (Figure 3A). After 24 h, biofilms were scraped and plated on selective agar. This demonstrated significantly fewer CFUs of UHW3 at 32% (w/v) and 64% (w/v) honey in comparison to 2% (w/v) honey determined by counting colony forming units (*p* = 0.0281 and 0.0152 respectively) (Figure 3B). Figure 3C shows that for PA14 there were fewer CFUs at 32% (w/v) and 64% (w/v) honey (*p* = 0.0033 and *p* = 0.0096).

### Honey scaffolds do not impact the biofilm growth of UHW3 and PA14 co-cultures

After establishing that Manuka honey inhibited both planktonic and biofilm formation of UHW3 and PA14 we wanted to determine if this activity would be retained when embedded in a dermal regeneration template composed of PCL, gelatin, and Manuka honey. Three types of scaffolds were created: a control containing PCL and gelatin (PG); a PCL, gelatin and 10% honey scaffold (PGH10); and a PCL, gelatin and 20% honey scaffold (PGH20). Previously, scanning electron microscopy of PCL-honey scaffolds demonstrated pore sizes between 50–100 um and fiber diameters between 2–3 um (Hilliard et al., 2019). The addition of gelatin resulted in similar scaffolds with non-woven and randomly oriented fibers extending in all directions. The addition of gelatin created scaffolds with similar pore sizes and fiber diameters between 1–2 um (data not shown).

**Figure 4:**
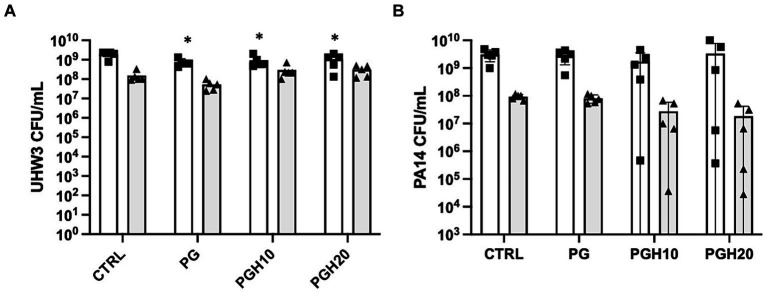
Honey scaffolds do not impact the growth of UHW3 and PA14 biofilm co-cultures. UHW3 and PA14 biofilms were established at a ratio of 250:1 in a 24-well plate with honey scaffolds and incubated for 24 h at 37°C. The colony forming units for **(A)** UHW3 and **(B)** PA14 were determined by performing a series of 1/10 dilutions and plating each dilution on selective MSA plates or PSA plates, respectively (*n* = 3). Error bars are represented as standard deviation. White bars with squares represent planktonic CFUs, and shaded bars with triangles represent biofilm CFUs. A two-way anova with Tukey’s multiple comparison was used to determine significance at an alpha level of 0.05. An asterisk indicates significance (*p* < 0.05).

Circular PG, PGH10 and PGH20 scaffolds (10 mm in diameter) were placed in wells containing UHW3 and PA14 (250:1). After 24 h, bacterial growth in response to the scaffolds was assessed. The supernatant was removed and plated on selective agar to measure growth of planktonic bacteria. The biofilm was scraped off the sides of the wells, resuspended in PBS, and plated on selective agar. Both the PG control and honey-embedded scaffolds significantly reduced planktonic UHW3 in comparison to the no scaffold control (*p* < 0.0001, *p* = 0.0004, and *p* = 0.0029 for PG, PGH10, and PGH20 respectively) but did not impact the biofilm growth of UHW3 (Figure 4A). The honey scaffolds had no impact on either planktonic or biofilm PA14 in comparison to the no scaffold control and the PCL-gelatin scaffold (Figure 4B).

### Honey scaffolds promote AgrA expression in UHW3/PA14 co-cultures

**Figure 5:**
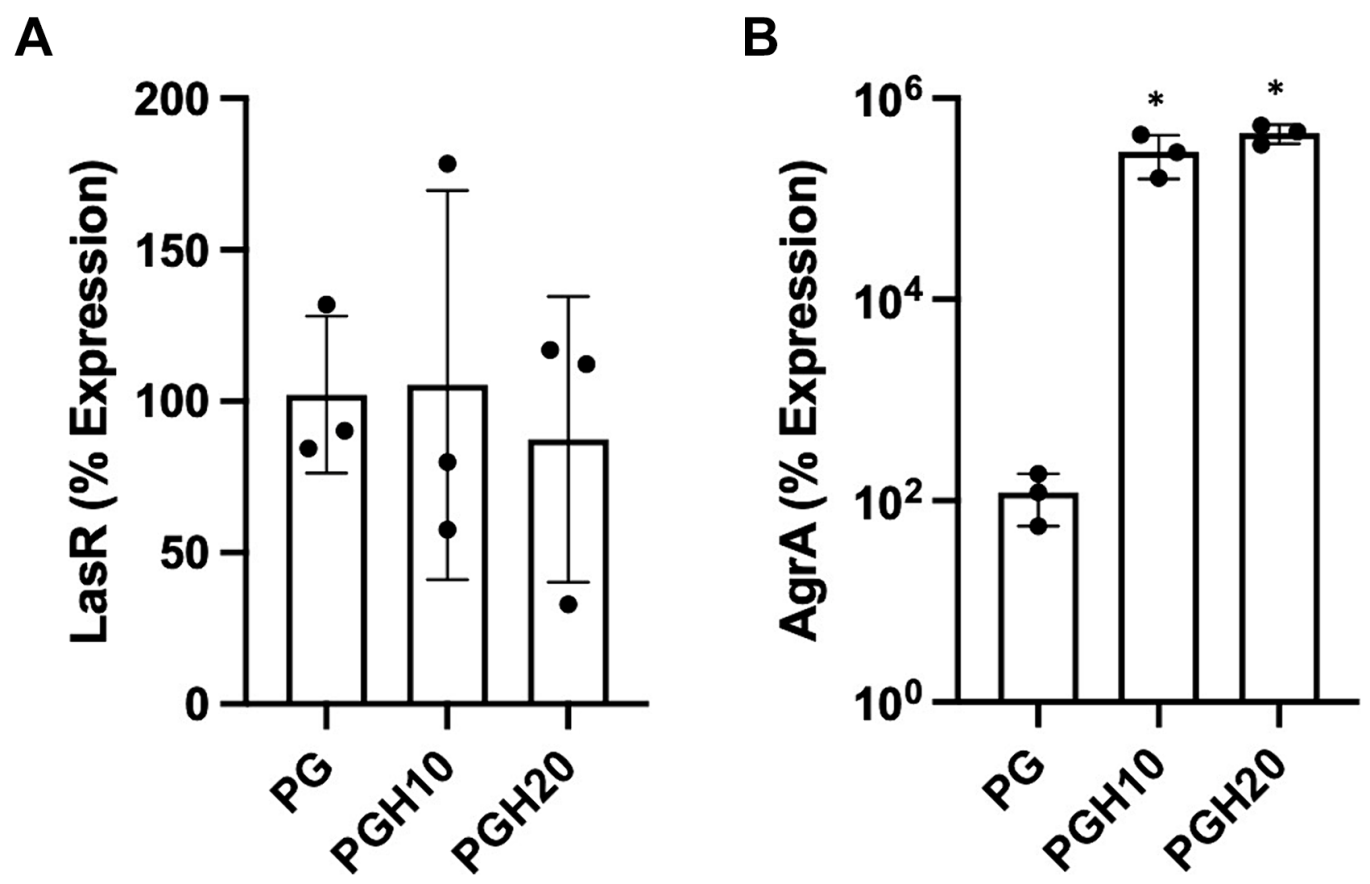
Honey scaffolds promote the expression of AgrA. UHW3 and PA14 were seeded at a ratio of 250:1 in a 24 well plate with honey scaffolds and incubated for 24 h at 37°C. Gene expression for **(A)** AgrA, and **(B)** LasR were determined by real-time PCR (*n* = 3). Error bars are represented as standard deviation. A one-way Anova Tukey test was used to determine significance at an alpha level of 0.05. An asterisk indicates significance (*p* < 0.05).

Figure 5 shows gene expression of LasR and AgrA as determined by real time PCR. The LasR gene is involved in *P. aeruginosa* quorum sensing while AgrA is involved in *S. aureus* biofilm formation. Figure 5A shows that the honey scaffolds had no impact on the expression of LasR. In contrast, Figure 5B demonstrates that the honey scaffolds significantly increased the expression of AgrA compared to scaffolds without honey (*p* = 0.0226 and *p* = 0.0031 for PGH10 and PGH20 respectively).

## Discussion

The antibacterial properties of Manuka honey can be attributed to the high osmolarity of the sugar component, low pH, and antibacterial components such as methylglyoxal (McLoone et al., 2016b). Honey has been tested against an extensive range of pathogens, including *Staphylococcus* and *Pseudomonas*, both in planktonic and biofilm form (Alandejani et al., 2009; Albaridi, 2019; Lu et al., 2019). While the concentration of honey required to inhibit growth differs between studies, the antibacterial actions of honey are well documented and substantial. For example, one study demonstrated that 16% (w/v) Manuka honey inhibited both the planktonic and biofilm growth of PA14. Surprisingly, a 16% sugar solution was also able to inhibit PA14 suggesting this strain has some tolerance to the methylglyoxal component of Manuka honey (Lu et al., 2019). For established *P. aeruginosa* biofilms, 32% (w/v) honey was required to significantly reduce established biofilm biomass (Lu et al., 2019). In *S. aureus* cultures, concentrations as low as 5% (w/v) inhibited growth and promoted loss of cell viability (Jenkins et al., 2011b). Based on the substantial data demonstrating that Manuka honey can inhibit diabetic wound pathogens, we hypothesized Manuka honey would inhibit the planktonic and biofilm growth of methicillin-resistant *S. aureus* strain UHW3 and *P. aeruginosa* strain PA14.

The growth response data for both UHW3 and PA14 demonstrated that honey can exhibit bacteriostatic and bactericidal effects on these pathogens. In general it required lower concentrations of honey to inhibit and kill UHW3 (14–22% (w/v) and 22–24% (w/v) respectively) compared to PA14. No concentration of honey tested was bactericidal against PA14, however there were bacteriostatic effects. Future studies with PA14 should test concentrations higher than 26% to achieve total inhibition of *P. aeruginosa,* although this concentration would be difficult to achieve in a dermal regeneration template. The growth of planktonic bacteria and biofilms were inhibited compared to the control at ≥20% and ≥ 16% (w/v) Manuka honey, respectively (Figures 3, 4). Once a biofilm was fully established, however, Manuka honey had little impact on growth. In fact, concentrations as high as 64% (w/v) had no significant effect on the viable colonies of UHW3 or PA14 in comparison to the no honey control.

Culture media may have a substantial impact on *S. aureus* and *P. aeruginosa* growth and biofilm formation. Previous studies utilizing Nutrient Broth, Brain Heart Infusion (BHI) broth, Luria-Bertani broth, or RPMI 1640 medium showed that the highly nutritious BHI promoted robust biofilm formation for *S. aureus* and *P. aeruginosa* co-cultures and is used for generating biofilms (Noleto et al., 1987; Singh et al., 2017; Wijesinghe et al., 2019). In the present study we used BHI, in contrast to many bacterial studies done using Mueller Hinton agar and broth. This may explain the need for high concentrations of honey needed to kill or inhibit bacterial growth in our studies.

While the use of honey for wound healing has a long history, it is difficult to apply and may leak out of the wound at body temperature. Previously, we included honey in slow release dermal regeneration templates to allow for a more efficient delivery of honey to the wound bed. These PCL-honey scaffolds served to provide a three-dimensional structure for cells to enter and re-epithelialize the wound (Hilliard et al., 2019). To extend our previous studies we developed novel PCL, gelatin, honey scaffolds and tested their antibacterial properties. We hypothesized the scaffolds containing 10% (w/v) and 20% (w/v) honey would inhibit the growth of Methicillin-resistant *S. aureus* and *P. aeruginosa* co-cultures; however, we did not find that scaffolds containing either 10% (w/v) or 20% (w/v) honey impacted the growth of UHW3 or PA14 in either planktonic or biofilm form at 24 h. Previous studies showing that low concentrations of honey (~5%) could inhibit the growth of *S. aureus* were performed at early times between 60–1,440 min and this may be one explanation for the difference (Jenkins et al., 2011b). Additionally, embedding Manuka honey into PCL-gelatin scaffolds presents a unique fabrication challenge. Concentrations greater than 20% are difficult to incorporate into PCL-gelatin scaffolds as the high viscosity of the solution can inhibit the electrospinning process. Therefore, in the present study it was only feasible to incorporate up to 20% honey into PCL-gelatin scaffolds with the fabrication technique utilized. Furthermore, we demonstrated with MIC and MBIC data that concentrations ≥14% are required to inhibit and/or kill pathogens with a bolus dose of Manuka honey. As such, the slow release of lower concentrations of Manuka honey (≤20%) from scaffolds are not sufficient in this study to inhibit growth. Interestingly, we observed fewer viable colonies of planktonic UHW3 after exposure to all scaffold conditions in comparison to the no scaffold control (Figure 4); this did not appear to be a honey effect but rather a scaffold effect. It will be important to investigate further whether the scaffold promotes attachment of either UHW3 or PA14, which could impact the relative quantities of planktonic bacteria in liquid suspension. This could potentially explain why the honey scaffolds promoted more UHW3 biofilm biomass in the wells compared to PA14, and why UHW3 planktonic bacteria slightly declined in the presence of the scaffold.

In addition to examining the impact of the honey-embedded scaffolds on bacterial growth, we next determined the effect on the expression of two quorum sensing genes, LasR and AgrA. Real-time PCR of PA14 demonstrated the honey scaffolds had no impact on LasR expression. This was in contrast to reports suggesting concentrations as low as 1% honey can cause a substantial reduction in LasR expression (Ahmed and Salih, 2019). Of note, Pesci et al. found that LasR expression is upregulated during the last half of log-phase growth, and a study by Ahmed et al. was performed during mid-exponential growth phase (Ahmed and Salih, 2019; Pesci et al., 1997). The lack of effect in the present study may be because samples were obtained 24 h after bacteria were exposed to the scaffold, when bacteria have passed the exponential growth phase and are in a stationary or death phase.

AgrA is a response regulator found on the Agr locus in *S. aureus*, which includes AgrB, AgrD, AgrC, and AgrA (Tan et al., 2018). Studies suggest that the *agr* system is involved in biofilm detachment which increases the susceptibility of *S. aureus* to antibiotics (Boles and Horswill, 2008; Yarwood et al., 2004). In our study, UHW3/PA14 co-cultures were incubated with 10% (w/v) and 20% (w/v) scaffolds for 24 h. Both the 10% (w/v) and 20% (w/v) scaffold conditions significantly increased the expression of AgrA. The increased AgrA expression could suggest that honey exposure limits biofilm formation but our results with the honey-embedded scaffolds do not show an effect on biofilm formation. A previous microarray study examining a single culture of MRSA treated with 10% manuka honey (w/v) demonstrated a decrease in the Agr genes B, C, and D after four hours of honey exposure (Jenkins et al., 2014). The differences with our study may be due to differences in the exposure time or the fact that our bacteria were in a co-culture with *P. aeruginosa*. Future studies should more thoroughly examine the time course of honey exposure on both individual and co-cultures.

In summary, diabetic wounds often contain biofilms composed of *S. aureus* and *P. aeruginosa.* The goal of this study was to determine the effect of manuka honey on *S. aureus* and *P. aeruginosa* biofilms. We found that single cultures of either *S. aureus* or *P. aeruginosa* were inhibited by manuka honey, but manuka honey PCL-gelatin scaffolds did not inhibit and effect on co-cultures of UHW3 and PA14; however, the scaffolds did increase expression of AgrA suggesting a potential effect on *S. aureus* biofilms.

## Data Availability

The original contributions presented in the study are included in the article/Supplementary material, further inquiries can be directed to the corresponding author.
