# Supplementary material for: PCL-gelatin honey scaffolds promote Staphylococcus aureus agrA expression in biofilms with Pseudomonas aeruginosa
Source: Front Microbiol. 2024 Sep 3;15:1440658. doi: 10.3389/fmicb.2024.1440658 (PMC11405313; doi:10.3389/fmicb.2024.1440658)
Supplement: Supplementary file 1 [file Presentation_1.pdf]

Supplementary Information

Methods

Zone of Inhibition

A sterile cotton tip was used to spread the diluted bacteria evenly over an MHA agar plate. Oxoid antibiotic discs (Thermo-fisher) were added to the plate and incubated overnight in 37°C. The zone of inhibition (ZOI) for *S. aureus* against cefoxitin (30µg), ciprofloxacin (5µg), gentamicin (10µg), tetracycline (30µg), erythromycin (15µg), and linezolid (10µg) was recorded. The ZOI for *P. aeruginosa* against piperacillin (30µg), ceftazidime (10µg), aztreonam (30µg), ciprofloxacin (5µg), and tobramycin (10µg) was recorded. Antibiotic sensitivities were determined by comparing the values to the European Committee on Antimicrobial Susceptibility Testing (EUCAST) breakpoint tables (Version 11).

Figure 1. Growth response of UHW3, 8532, PA14, and UHW3 to antibiotics. (A) The antibiotic susceptibility of UHW3 and 8532 to cefoxitin, ciprofloxacin, gentamicin, tetracycline, erythromycin, and linezolid (n=3). (B) The antibiotic susceptibility of PA14 and 13437 to piperacillin, ceftazidime, aztreonam, ciprofloxacin, and tobramycin (n=3).

Figure 2. Growth response of UHW3, 8532, PA14, and UHW3 Manuka honey. The growth response, represented as optical density, of UHW3, 8532, PA14, and 13437 to honey concentrations ranging from 15% (w/v) to 30% (w/v), measured at 600 nm (n=3). Error bars are represented as standard deviation. A two-way anova Tukey test was conducted to determine significance at an alpha level of 0.05. An asterisk indicates significance ($p<0.05$).

Figure 3. UHW3 and PA14 establish robust biofilms after 24 hrs. Colony forming units of UHW3 and PA14 (A) planktonic bacteria (B) biofilms in co-culture at a ratio of 250:1 after 4, 8, and 24 hours of incubation at 37°C (n=3). Error bars are represented as standard deviation. A two-way anova with Sidak's multiple comparison test was conducted to determine significance at an alpha level of 0.05. An asterisk indicates significance ($p<0.05$).
