# Supplementary figures and images for: PCL-gelatin honey scaffolds promote Staphylococcus aureus agrA expression in biofilms with Pseudomonas aeruginosa
Source: Front Microbiol. 2024 Sep 3;15:1440658. doi: 10.3389/fmicb.2024.1440658 (PMC11405313; doi:10.3389/fmicb.2024.1440658)

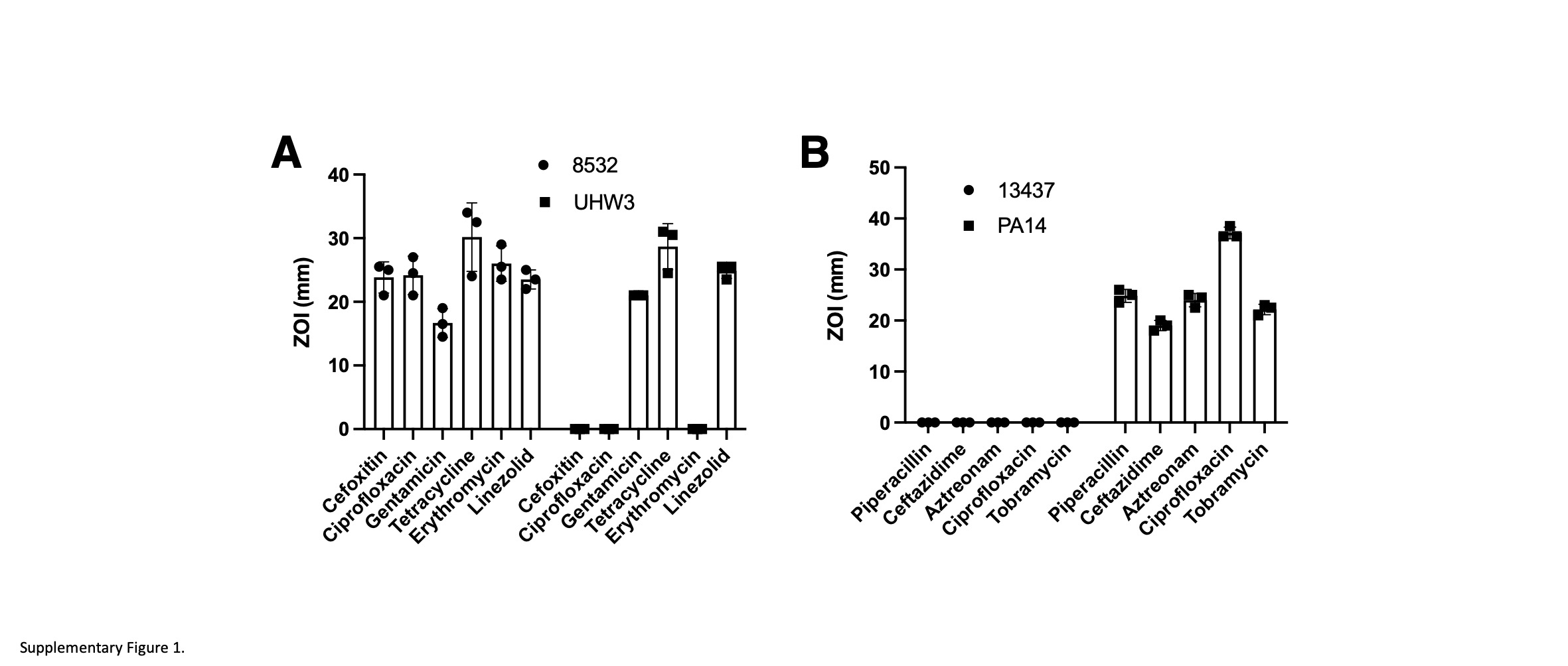

Supplement: Supplementary file 2 [file Image_1.JPEG]

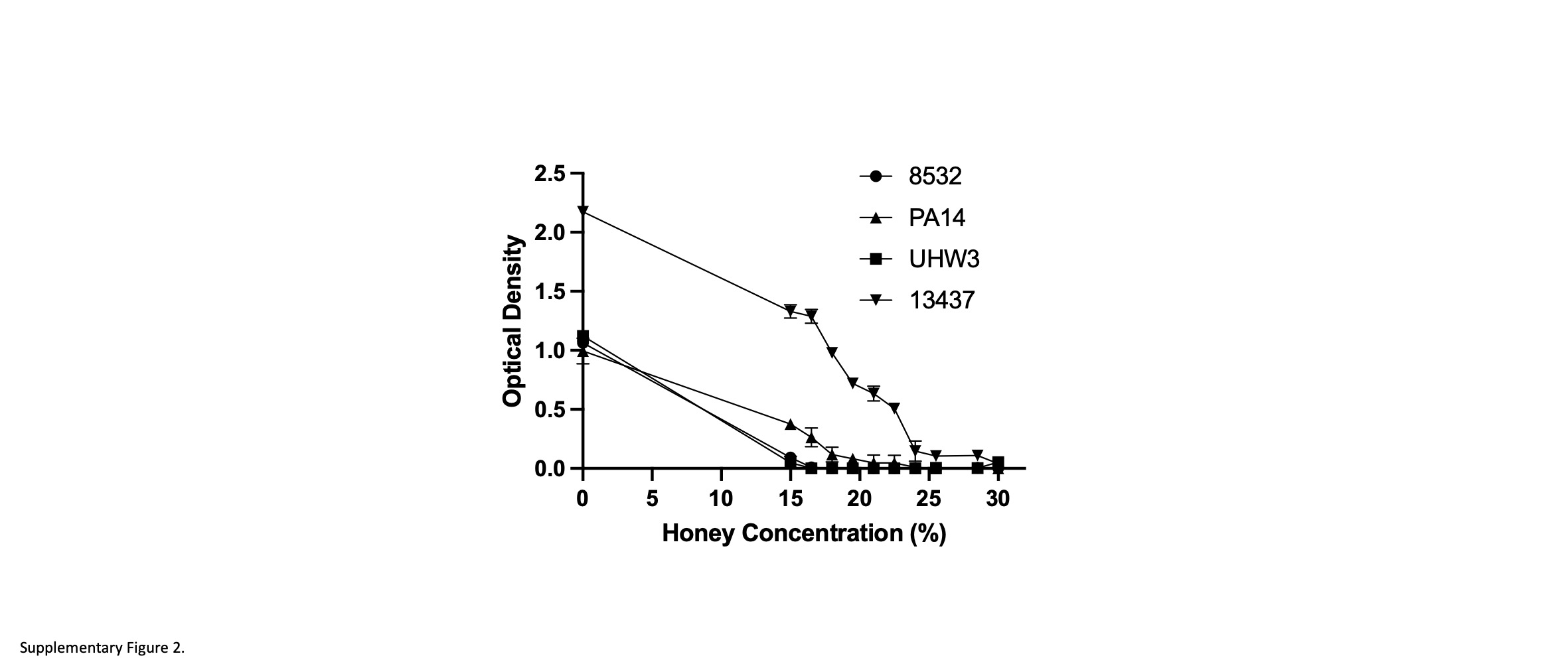

Supplement: Supplementary file 3 [file Image_2.JPEG]

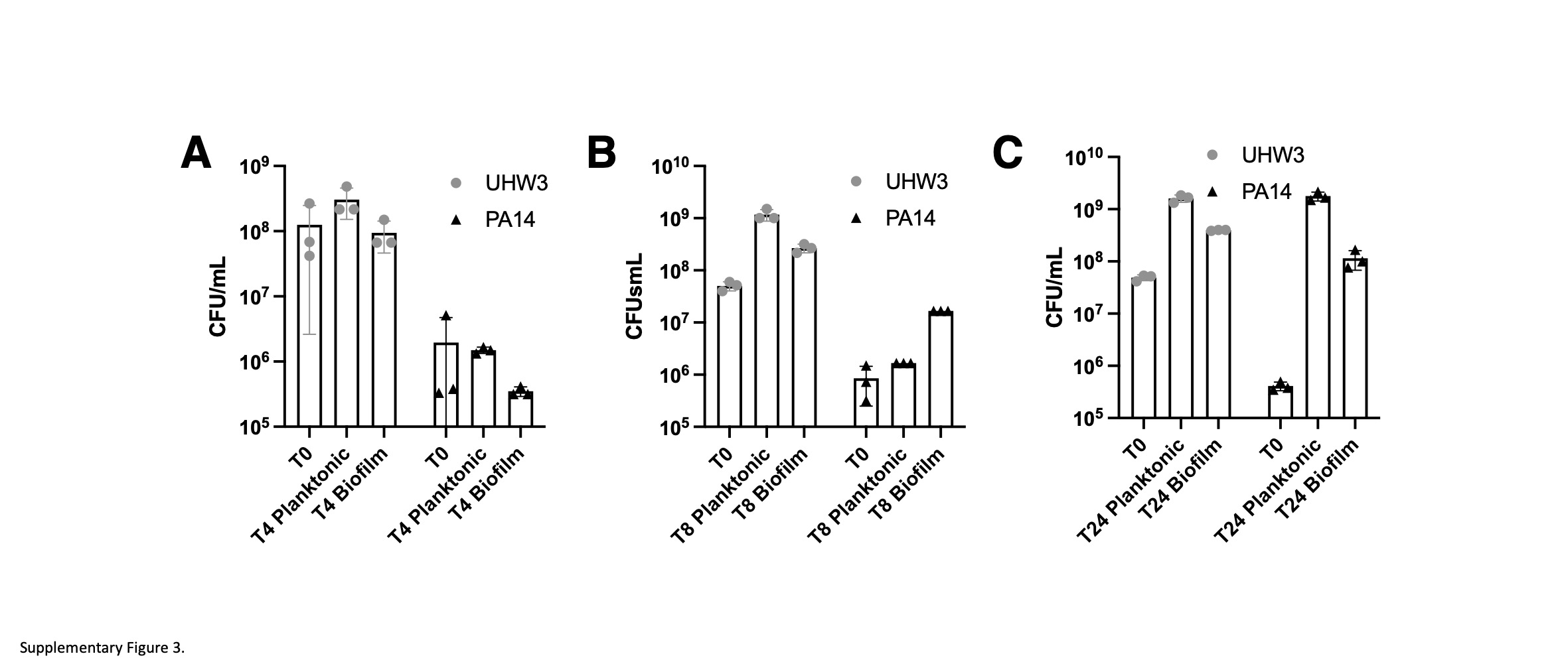

Supplement: Supplementary file 4 [file Image_3.JPEG]
